# Effects of Copper on Steroid Hormone Secretion, Steroidogenic Enzyme Expression, and Transcriptomic Profiles in Yak Ovarian Granulosa Cells

**DOI:** 10.3390/vetsci12050428

**Published:** 2025-04-30

**Authors:** Yanbing Lou, Tingting Yang, Chenglong Xia, Shijun Yang, Huidan Deng, Yanqiu Zhu, Jing Fang, Zhicai Zuo, Hongrui Guo

**Affiliations:** 1 College of Veterinary Medicine, Sichuan Agricultural University, Wenjiang, Chengdu 611130, China; louyanbing@stu.sicau.edu.cn (Y.L.); yangtingting@stu.sicau.edu.cn (T.Y.); xiachenglong@stu.sicau.edu.cn (C.X.); ysjsicau@hotmail.com (S.Y.); denghuidan@sicau.edu.cn (H.D.); zhuyanqiu@sicau.edu.cn (Y.Z.); 10310@sicau.edu.cn (J.F.); czyhzzc@126.com (Z.Z.); 2 Key Laboratory of Animal Diseases and Environmental Hazards of Sichuan Province, Sichuan Agriculture University, Wenjiang, Chengdu 611130, China

**Keywords:** steroid hormone, ovarian granulosa cells, yak, copper

## Abstract

Yak (Bos grunniens) is the main economic animal growing in the Qinghai–Tibet Plateau. Because of its poor growing environment, copper deficiency is common. Studies have shown that appropriate copper supplementation can improve the reproductive performance of heifers. In this study, the primary granulosa cells of yak were isolated, and different copper levels were established by adding copper chelator and copper sulfate. Biochemical, Western blot and transcriptome methods were used to reveal the mechanism of copper on the hormone secretion function of granulosa cells. The results showed that copper promoted hormone secretion by regulating the expression of steroid synthase such as StAR, CYP19A1 and CYP11A1. Transcriptome analysis showed that copper ion levels significantly affected the function of cells, and changes in copper ion level affected genes related to protein phosphorylation, lipid metabolism, lipid biosynthesis, steroid hormone synthesis and the MAPK pathway. In conclusion, copper can promote steroid hormone synthesis in the granulosa cells of yak follicles, and the MAPK signaling pathway may be involved.

## Simple Summary

Yak, as a unique species of plateau cattle, is highly susceptible to Cu deficiency, which is further exacerbated by poor soil conditions and low nutrient content in yak feed, which is associated with the decline of yak performance. Hormones synthesized by granulosa cells of yak follicles play an indispensable role in reproductive function. In this study, we investigated the effects of different concentrations of copper on the hormone secretion of cultured granulosa cells of yak follicles and the related molecular mechanisms. The above results showed that copper could positively promote the hormone secretion of female yaks, which provided a strategy direction with potential value for improving the fertility of yaks under the condition of copper deficiency.

## 1. Introduction

Copper (Cu) is an integral component of cellular physiology due to its unique chemical properties. Unlike most physiologically relevant metals, Cu accepts and donates electrons under physiological conditions and cycles between two oxidation states. Cu is an essential element in the synthesis of ceruloplasmin, which plays a key role in the metabolism of iron by helping to convert stored forms of iron into forms available for use by the body, thereby promoting iron absorption and utilization [1]. Cu is also a component of a variety of antioxidant enzymes, including Cu/Zn-superoxide dismutase (Cu/Zn-SOD, SOD1), which can remove superoxide radicals from the body and protect cells from oxidative stress damage [2].

Cu plays a very important role in the body of ruminants. It is an essential trace element for maintaining the normal physiological function, biochemical metabolism and the growth and development of ruminants. As early as the 1940s, hypocopper in cattle had been reported [3]. Today, copper deficiency in cattle is still common. In addition to the direct primary copper deficiency caused by insufficient copper intake, the main cause of copper deficiency in cattle is secondary copper deficiency caused by interference with copper absorption. Due to the interaction between Cu, molybdenum and sulfur (Cu-Mo-S) in the rumen of ruminants, the level of Cu absorption by ruminants is much lower than that of non-ruminants [4]. In western China, yaks develop a disease locally known as “sway disease” characterized by loss of appetite, diarrhea, pica, poor growth, emaciation, dyskinesia (walking slowly with slight signs of rock and shivering) and sway, which is caused by secondary copper deficiency [5]. Yaks in the northern Qinghai–Tibet Plateau of China have symptoms of secondary copper deficiency induced by molybdenum. Yaks with copper deficiency are emaciated and unstable, and the symptoms of copper deficiency are most serious during pregnancy and lactation [6]. From 2014 to 2019, copper deficiency occurred in almost all cattle farms in eastern and western Canada, with 20–40% of cows in each farm having lower serum copper content than normal, and the majority of such copper deficient cows showed symptoms of copper deficiency [7]. Copper deficiency can also cause morphological changes in bovine heart, resulting in significant damage to myocardium, high lipid oxidation level, and significantly reduced Cu-Zn superoxide dismutase activity [8].

It has been shown that adequate copper concentrations improve intracellular GSH content in oocytes and cumulus cells, the DNA integrity of cumulus cells during in vitro maturation of bovine oocytes, and the development of preimplantation embryos [9]. Additionally, Cu may promote the secretion of luteinizing hormone-releasing hormone from isolated hypothalamic granules [10,11]. Copper deficiency can indirectly affect reproductive processes by impairing bone development and keratinization, reducing antioxidant capacity in cows, and impacting angiogenesis [12,13]. In 1936, researchers found that ovulation could be induced in model animals by injection of copper salts [11]. Cu can maintain the erratic activity of pituitary hormones in the blood and also promote the release of gonadotropin-releasing hormone and luteinizing hormone. Studies have shown that Cu^2+^ levels in follicular fluid affect steroid hormone production by follicular granulosa cells [14]. Cu can also affect the normal development and ovulation of follicles. Studies have shown that the number of follicles in the ovary is significantly reduced by short-term high-dose Cu treatment, and long-term high-dose Cu treatment can affect the whole ovary [15]. In the reproductive system of female mammals, oocytes, as female reproductive cells, have become important experimental subjects in reproductive and developmental biology research due to their unique biological characteristics. Granulocytes (GCs) play multiple key roles in the follicular microenvironment: on the one hand, they support follicular growth by providing nutrients and bioactive factors, and on the other hand, they regulate the maturation process of oocytes by synthesizing reproductive hormones such as estradiol (E2) and progesterone (P4) [16]. From the perspective of cellular interaction, GCs coordinate signal communication between oocytes and follicular membrane cells through their proliferative activity and endocrine function, thereby participating in the regulation of various stages of follicular development [16]. Jiang et al. further confirmed that GCs are not only an ideal cell model for studying the regulation of follicle development by steroid hormones, but also a core regulator for maintaining the stability of the follicular environment and promoting oocyte development [17]. Our previous studies have shown that Cu^2+^ can induce steroid hormone secretion in BGCs (Bos grunniens granlosa cells), but the specific mechanism remains unclear.

The aim of this study is to elucidate the mechanism by which Cu^2+^ affects the secretion of granulosa cell hormones. Quantitative real-time PCR, Western blotting and transcriptome analysis were used to elucidate the effect of Cu^2+^ on steroid hormone synthesis in BGCs and the underlying molecular mechanisms. The results of this study may provide new insights and theoretical support for addressing reproductive disorders associated with copper deficiency in yaks.

## 2. Materials and Methods

### 2.1. Materials

Cu sulfate (CuSO_4_; CAS No. 7758-98-7; AR 99%) was sourced from Chengdu Kelong Chemical Co., Ltd. (Chengdu, China). Ammonium tetrathiomolybdate (TTM; CAS No. 15060-55-6) was obtained from Sigma Aldrich (St. Louis, MO, USA). Phenylmethanesulfonyl fluoride (PMSF; CAS No. ST506), Nonidet P-40 (NP40) lysis buffer (Cat No. P0013F), BeyoECL Star (Cat No. P0018AM), and bicinchoninic acid (BCA) protein assay kit (Cat No. P0012) were purchased from Beyotime Biotechnology (Shanghai, China). Estradiol (E2) Enzyme-linked immunosorbent assay (ELISA) kits (Cat No. MM-0023O1) and progesterone (P4) ELISA kits (Cat No. MM-50918O1) were sourced from Mmbio (Jiangsu, China). Follicle-stimulating hormone (FSH; Cat No. F8470) was obtained from Solarbio (Beijing, China), and testosterone (Cat No. T102169) was obtained from Aladdin (Shanghai, China). Superoxide Dismutase (SOD) typed assay kit (Cat No. A001-2-2) was obtained from Nanjing Jiancheng Bioengineering Institute (Nanjing, China).

The antibodies used in this study included: anti-rabbit StAR (bs-3570R, Bioss, Beijing, China, 1:500), anti-rabbit CYP19A1 (ET1703-77, HuaBio, Zhejiang, China, 1:1000), anti-rabbit CYP11A1 (13363-1-AP, HuaBio, 1:1000), anti-rabbit HSD3B1 (HA500082, HuaBio, 1:1000), anti-rabbit HSD17B1 (ER1910-88, HuaBio, 1:1000), anti-rabbit LOX (ET1706-31, HuaBio, 1:1000).

### 2.2. Cell Culture and Treatments

Ovarian tissues from yaks (*n* = 18; aged 3 to 4 years) were procured from a slaughterhouse located in Chengdu. BGCs were extracted with reference to previous studies [18]. The cells were seeded into T25 culture flasks after adjusting the concentration to 5 × 10^6^ and then incubated in a cell incubator at 37 °C and 5% CO_2_. In subsequent experiments aimed at investigating alterations in steroidogenesis within BGC cultures, the BGC were cultured in a basal medium (DMEM/F-12 containing 1% penicillin-streptomycin-amphotericin B and 10% FBS) supplemented with 20 ng/mL testosterone and 30 mIU of follicle-stimulating hormone (FSH) [19,20]. TTM is a high-affinity copper complex with excellent safety qualities. It forms a stable three-way complex with serum albumin and Cu^2+^, so that the complex Cu^2+^ cannot be used by cells, and is often used to chelate intracellular Cu^2+^ [21]. In this trial, low Cu^2+^ status was determined by TTM and SOD1 activity was used as a marker of Cu^2+^ status [22]. A higher Cu^2+^ state was established by additional supplementation of CuSO_4_. BGCs were divided into five groups: control group, TTM group (TTM 10, 20 μmol), CuSO_4_ group (CuSO_4_ 150, 300 μmol). They were treated for 12 and 24 h, respectively.

### 2.3. Cellular Cu Assay

Cell samples and media were collected and quantified utilizing a cell counting instrument (IC1000; Countstar, Shanghai, China), followed by analysis through inductively coupled plasma mass spectrometry (ICP-MS). The samples underwent digestion with 69–70% nitric acid at a temperature of 95 °C for a duration of 2 h. Subsequent to digestion, the samples were filtered using polytetrafluoroethylene membrane filters and subsequently analyzed with an Agilent 7800 ICP-MS system (Agilent Technologies, Inc., Santa Clara, CA, USA).

### 2.4. Transcriptome Sequencing and Transcriptomic Analysis

BGCs were divided into three groups: a control group, a TTM group (TTM at 20 μmol), and a Cu group (CuSO_4_ at 300 μmol), with three biological replicates per group. Cell samples were collected 24 h post-treatment. Sequencing and analysis were conducted by Novogene Ltd. (Beijing, China). RNA was extracted from the cells using standard protocols, and RNA integrity was assessed using an Agilent 2100 Bioanalyzer. Complementary DNA (cDNA) libraries were constructed with the Illumina TruSeq RNA Sample Preparation Kit (Illumina, CA, USA). All samples were aligned with the bovine genome (http://ftp.ensembl.org/pub/release-105/fasta/bos_taurus/) (accessed on 12 June 2024) to meet library screening requirements. Principal component analysis (PCA) was performed based on the gene expression values (FPKM) across all samples. Differentially expressed genes (DEGs) between groups were identified using a significance threshold of *p* ≤ 0.05. Following differential gene analysis, Gene Ontology (GO) and Kyoto Encyclopedia of Genes and Genomes (KEGG) enrichment analyses were performed using the ClusterProfiler R software (1.21.1.202) package. Genes associated with steroid hormones and the MAPK signaling pathway were selected based on enrichment results. Protein–protein interaction (PPI) networks were constructed using the STRING database (https://cn.string-db.org/) (accessed on 13 September 2024) with Homo sapiens protein interaction data, considering interactions with scores above the 0.7 threshold as significant. The resulting PPI networks were visualized using Cytoscape software (3.10.3).

### 2.5. Cells SOD1 Activity Assay

After BGCs were treated with CuSO_4_ or TTM for 12 and 24 h, cells were washed 1–2 times with precooled saline on ice or at 4 °C. The precipitate was homogenized in precooled saline at 4 °C and collected in a centrifuge tube. Then, the collection was centrifuged at 4 °C at a speed of 12,000 r/min, and the resulting supernatant was detected according to the SOD1 genotyping assay kit instructions.

### 2.6. Cell Activity Assay

BGCs were cultured in 96-well plates until they reached approximately 60% confluence. Following this, the culture medium was replaced with varying concentrations of CuSO_4_ and TTM, and the cells were treated for either 12 or 24 h. Upon completion of the treatment, the wells were rinsed two to three times with phosphate-buffered saline (PBS). Subsequently, 90 μL of fresh medium and 10 μL of MTT (Methylthiazolyldiphenyl-tetrazolium bromide) solution (M1020, Solarbio, Beijing, China) were introduced into each well, and the incubation continued for a duration of 4 h. At the conclusion of the incubation period, the supernatant was removed, and 110 μL of formazan solution was added to each well. The plate was then gently agitated at a low speed for 10 min to ensure complete dissolution of the resulting crystals. Finally, the absorbance of each well was quantified at a wavelength of 490 nm using an enzyme-linked immunoassay reader.

### 2.7. ELISA

The treated cell culture medium was collected, and the clear supernatant was isolated through centrifugation at speeds ranging from 2000 to 3000 RPM. Estradiol (E2, MM-0023O1) and progesterone (P4, MM-50918O1) were sourced from Mmbio in China and were allowed to equilibrate at room temperature prior to their application. Samples, standards, and blank controls were established, and precoated microtiter wells were prepared accordingly. The specimens, standards, and horseradish peroxidase (HRP)-labeled assay antibodies were sequentially added, followed by incubation at elevated temperatures and a thorough washing procedure. The colorimetric reaction was initiated by the substrate TMB (3, 3′,5,5′-Tetramethylbenzidine), which exhibits a blue color in the presence of peroxidase and transitions to yellow upon the addition of an acidic solution. The intensity of the resulting color is directly proportional to the concentration of E2 or P4 present in the sample. The absorbance of each well was measured at a wavelength of 450 nm utilizing an enzyme-linked immunoassay reader.

### 2.8. Western Blotting

BGC was cleaved with NP40 and 1% phenylmethane sulfonyl fluoride (PMSF). Lysate supernatants were collected and protein concentrations were quantified using the BCA Protein Assay kit. Subsequently, the separated protein samples were separated by SDS-PAGE and then transferred to PVDF membranes. Finally, ImageJ2x software was used to analyze significant changes in protein expression.

### 2.9. Quantitative Reverse Transcription PCR (RT q-PCR)

After drug treatment with BGCs, total RNA was extracted using an animal total RNA isolation kit (RE-03014, Foregene, Chengdu, China). RNA was reverse transcribed into complementary DNA (cDNA) using the PrimeScript^®^RT Kit (RR047A, Takara, Tokyo, Japan). Primers were designed and synthesized by Sangyo Bioengineering (Shanghai, China) Ltd., as detailed in Table 1. CT values were obtained using SYBR PRIME qPCR (FASTHS) (BG0014, Baoguang, Chongqing China) after predenaturation, denaturation, annealing, and extension. The amplification was monitored using the LightCycler^®^480 Real-Time PCR System (Bio-Rad, Hercules, CA, USA), with the β-actin gene employed as an internal control. The quantification of mRNA expression levels was performed using the 2−ΔΔCT method.

### 2.10. Statistical Analysis

Data analysis was performed using GraphPad Prism 8.0 and SPSS software (version 21.0, IBM Corporation, Armonk, NY, USA). Independent samples *t*-tests and one-way analysis of variance (ANOVA) were employed to compare data across different groups. The results are presented as means ± standard deviation, with a *p*-value of less than 0.05 indicating statistical significance.

## 3. Results

### 3.1. Copper Status Affects Hormone Secretion in Ovarian Granulosa Cells of Yak

**Figure 1:**
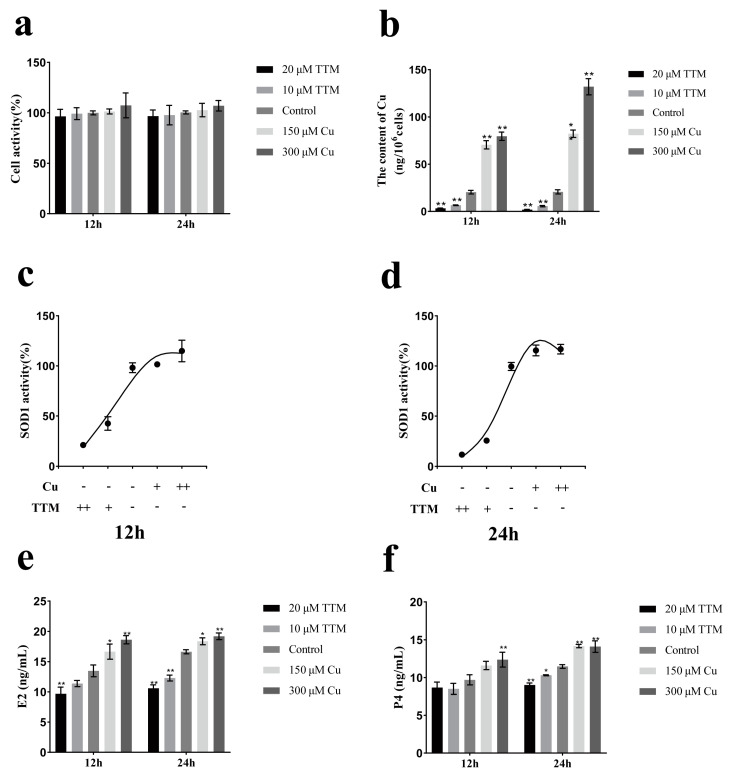
Copper status affects hormone secretion in ovarian granulosa cells of yak. Changes in cell viability (**a**), cellular copper concentration (**b**), E2 (**e**) and P4 (**f**) of yak ovarian granulosa cells at different copper status. Effects of different copper concentrations on SOD1 activity of follicular granulosa cells in yaks after 12 h (**c**) and 24 h (**d**) treatment. Data are presented with the means ± standard deviation (*n* = 3). In (**a**–**f**), data are presented as mean ± standard error and statistically analyzed using ANOVA with *t*-tests. Compared with the control group, * *p* < 0.05, ** *p* < 0.05.

To investigate the effect of Cu^2+^ levels on hormone secretion in BGCs, various concentrations of CuSO_4_ were added to the culture medium, and TTM was introduced to sequester Cu^2+^. As depicted in Figure 1a–d the cellular copper concentration and activity of SOD1 increased with rising Cu^2+^ concentrations, with no significant impact on cell viability. Conversely, the addition of TTM resulted in decreased SOD1 activity, while cell viability remained unchanged. Figure 1e,f show that the concentrations of estradiol and progesterone in the culture supernatant increased with increasing Cu^2+^ concentrations and decreased with decreasing Cu^2+^ concentrations at different Cu^2+^ states.

### 3.2. Copper Promoted Steroid Synthetase Gene and Protein Expression in Granulosa Cells of Yak

**Figure 2:**
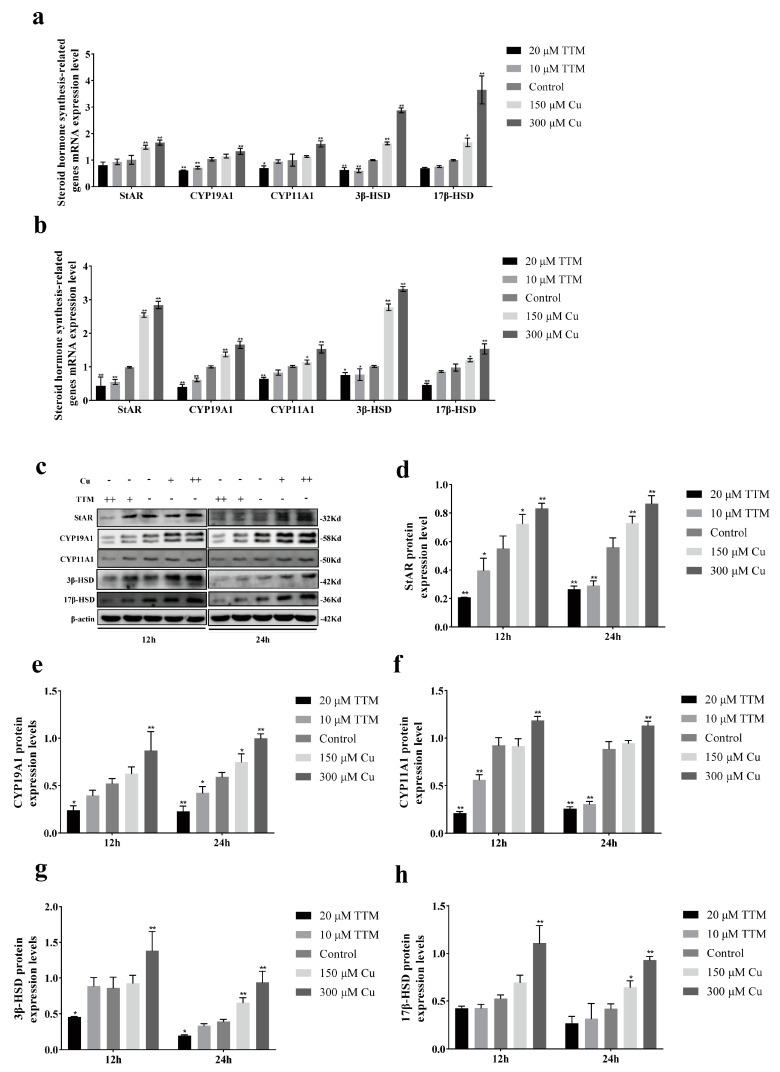
Copper promotes genes related to steroid hormone synthesis in yak follicular granulosa cells. Effect of different Cu^2+^ concentrations on steroid hormone synthetase gene expression levels in granulosa cells of yak follicles after 12 h (**a**) and 24 h (**b**) treatment. (**c**) Western blot analysis of StAR, CYP19A1, CYP11A1, 3β-HSD and 17β-HSD. (**d**–**h**) Quantification of StAR, CYP19A1, CYP11A1, 3β-HSD and 17β-HSD protein expression in yak ovarian granulosa cells after 12 h and 24 h of treatment with different Cu^2+^ status. Cu+: 150 μm CuSO_4_; Cu++; 300 μm CuSO_4_; TTM+; 10 μm TTM; TTM++; 20 μm TTM. Data are presented with the means ± standard deviation (*n* = 3). In (**a**,**b**,**d**–**h**), data are presented as mean ± standard error and statistically analyzed using ANOVA with *t*-tests. Compared with the gene silencing group, * *p* < 0.05, ** *p* < 0.01.

To further investigate the impact of Cu^2+^ levels on steroid hormone secretion in BGCs, we assessed the mRNA and protein expression levels of these five genes 12 and 24 h after TTM and CuSO_4_ treatments, respectively. The results indicated that, compared to the control group, the protein and mRNA expression levels of steroid hormone synthesis-related genes StAR, CYP19A1, CYP11A1, 3β-HSD, and 17β-HSD significantly increased with higher Cu^2+^ concentrations (*p* < 0.05) (Figure 2). Specifically, after 12 and 24 h of treatment, the StAR mRNA expression in the high copper group (300 μM CuSO_4_) increased by 1.8-fold and 2.5-fold, respectively, while protein levels increased by 2.1-fold and 3.0-fold, respectively (Figure 2a,b,d). Similarly, the mRNA expression of 3β-HSD increased by 2.2 times at 24 h, but its protein level increased more significantly (3.5 times) (Figure 2b,g). In contrast, the gene and protein expression of the copper chelator TTM treatment group (20 μM) were significantly lower than those of the control group, indicating that copper deficiency inhibits the expression of key enzymes in the steroid hormone synthesis pathway.

### 3.3. Transcriptomic Analysis of Copper Status in Ovarian Granulosa Cells Function

Based on the aforementioned findings, in order to further elucidate its molecular regulatory mechanism, this section uses transcriptome sequencing technology to systematically compare the global gene expression changes in cells under different Cu^2+^ states. Through differential expression gene analysis, functional enrichment, and protein interaction network construction, the aim is to reveal the key signaling pathways (such as the MAPK pathway) and potential target genes involved in Cu^2+^ regulation of steroid hormone synthesis, providing a theoretical basis for a deeper understanding of the molecular basis of copper deficiency induced reproductive disorders in yaks.

**Figure 3:**
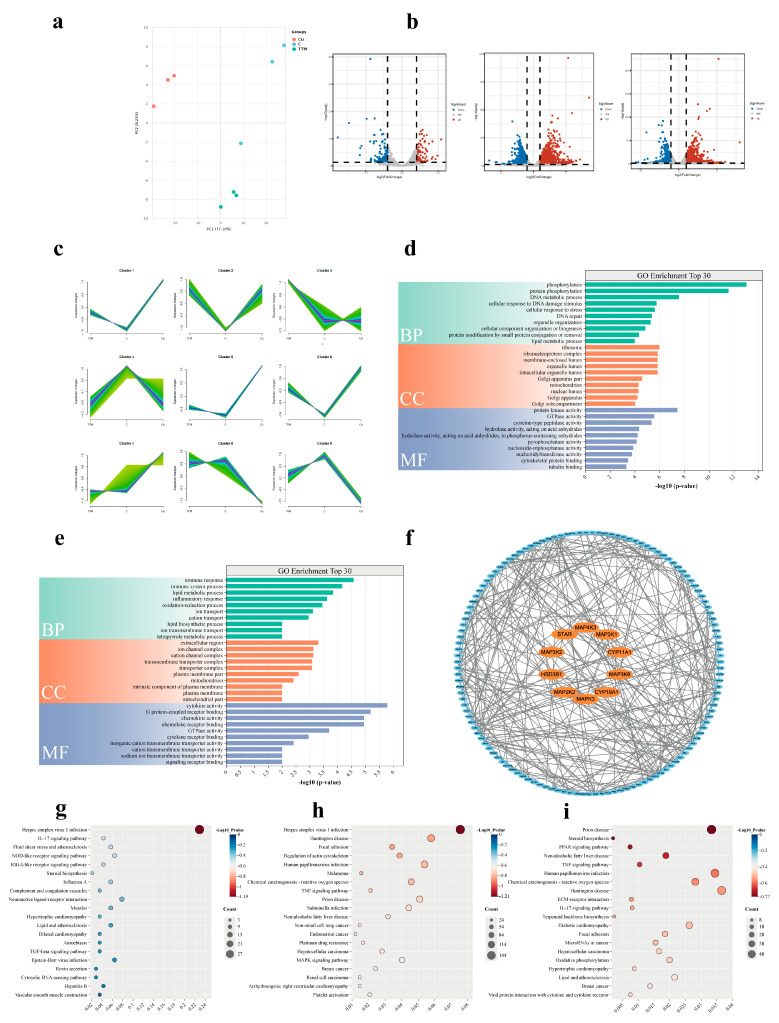
Transcriptomic analysis of copper status in ovarian granulosa cell function. (**a**) Results of the Principal Component Analysis (PCA) of gene expression data from BGCs based on fragments per kilobase million. (**b**) Volcano plots were used to illustrate changes in gene expression in the TTM and Cu groups versus the control group. (**c**) Mfuzz clustering of TTM group, Cu group and control groups. (**d**) The annotation of the first 30 GO functional DEGs between the Cu group and control group. (**e**) The annotation of the first 30 GO functional DEGs between the Cu group and TTM group. (**f**) Analysis of protein–protein interaction network on steroid hormone secretion related genes and MAPK signaling pathway-related genes. (**g**–**i**) Bubble plots of the top 20 enriched KEGG pathways in differentially expressed genes in the Cu group, TTM group and control groups. “Count” indicates the number of DEGs which were annotated to the corresponding pathway: a bigger value indicates that more DEGs were enriched in this pathway; “Gene Ratio” indicates the ratio of the number of DEGs which was annotated to the corresponding pathways to the total number of DEGs: a bigger value indicates a higher enrichment level of DE Metas in this pathway; and “Adjusted *p*-values” indicates the significance corrected through multiple hypotheses.

PCA demonstrated that sample replicates clustered closely together, indicating good reproducibility for future analyses (Figure 3a). As shown in Figure 3b, a total of 213 genes were up-regulated, while 94 genes were down-regulated in the TTM group compared to the control group (*p* < 0.05). In the Cu group, 2773 genes were up-regulated, and 1973 genes were down-regulated compared to the control group (*p* < 0.05). Additionally, a comparison between the Cu group and the TTM group revealed that 1168 genes were up-regulated, while 863 genes were down-regulated. To better illustrate the alterations in gene expression in response to varying Cu^2+^ concentrations, we conducted a cluster analysis on the gene expression profiles of all samples, revealing that the trends in gene expression changes in BGCs correlated with increasing Cu^2+^ concentrations could be classified into nine distinct categories. Notably, the gene expressions of StAR, CYP19A1, CYP11A1, and 3β-HSD exhibited significant increases with elevated Cu^2+^ levels (Figure 2c).

Functional enrichment analysis of DEGs for biological processes, cellular components, and molecular functions was performed on the DEG datasets from the Cu and control groups, yielding 175 annotation results with a *p*-value of less than 0.05. The first 30 annotations are illustrated in Figure 3e. The DEGs associated with biological processes primarily involved protein phosphorylation, lipid metabolism, and DNA metabolism. In terms of cellular components, DEGs were predominantly concentrated in ribosomes, ribosomal protein complexes, and membrane-enclosed cavities. Furthermore, DEGs in the molecular function category were mainly associated with protein kinase activity, GTPase activity, and cysteine-type peptidase activity. Additionally, functional enrichment analysis of biological processes, molecular functions, and cellular components was conducted for DEGs in the Cu and TTM group, resulting in 94 annotation results with a *p*-value of less than 0.05. The initial 30 annotations are depicted in Figure 3d. DEGs associated with biological processes were primarily involved in immune responses, lipid metabolic processes, and lipid biosynthesis. In the cellular components category, DEGs were predominantly located in extracellular regions, ionic pore complexes, and cation channel complexes. Moreover, DEGs in the molecular function category were primarily associated with binding to G protein-coupled receptors, which are linked to cytokine and chemokine activities. As illustrated in Figure 3f, genes associated with steroid hormones and MAPK signaling pathways were selected based on the results of the enrichment analysis. A PPI network was constructed using the STRING database (https://cn.string-db.org/) (accessed on 13 September 2024) with data on Cattle sapiens’ protein interactions. Interactions with a score above the 0.7 threshold were considered significant, and the resulting PPI networks were visualized using Cytoscape software (3.10.3). PPI results showed that StAR, CYP19A1, CYP11A1, HSD3B1 and MAPK4K3, MAPK3K1, MAPK3K8, MAPK3, MAPK2K2 and MAPK3K2 were Hub genes.

KEGG enrichment analyses were performed on the TTM, control, and Cu groups, as shown in Figure 3g. This figure presents the top 20 KEGG pathways that exhibited the greatest enrichment in the TTM group compared to the control group. The TTM group demonstrated significant enrichment of DEGs in pathways such as herpes simplex virus 1 infection, the IL-17 signaling pathway, and steroid biosynthesis, among others. Additionally, the 20 KEGG pathways with the highest degree of enrichment in the Cu group compared to the control group are also depicted in Figure 3g. The DEGs in the Cu group were significantly enriched in pathways related to herpes simplex virus 1 infection, the TNF signaling pathway, the MAPK signaling pathway, and others. The top 20 KEGG pathways with the highest degrees of enrichment in both the Cu and TTM groups are presented in Figure 3g, with DEGs in the high Cu group predominantly enriched in pathways related to prion disease, steroid biosynthesis, and peroxisome proliferator-activated receptor (PPAR) signaling.

## 4. Discussion

Cu is an essential trace element for the growth and development of humans and animals. It is a cofactor for many proteins and enzymes. Due to their unique digestive system, ruminants have a much lower level of Cu absorption than non-ruminants [4]. Molybdenum induced secondary Copper deficiency is present in yaks from the northern Tibetan Plateau of China, and Copper deficiency is most severe during pregnancy and lactation. Granulosa cells are important participants in follicular development and female reproduction, and the steroid hormones estradiol and progesterone secreted by granulosa cells are essential for maintaining the reproductive process of female animals [23]. This study found that Cu promoted hormone secretion by regulating the expression of steroid synthetase, such as StAR, CYP19A1, 3β-HSD, etc. Transcriptome sequencing further revealed that Cu was significantly enriched in the MAPK signaling pathway. This study demonstrates the molecular mechanism of Cu regulating BGCs hormone secretion, and provides a new idea for the treatment of reproductive disorders related to copper deficiency in yaks.

Granulosa cells can provide a large number of substances and growth factors for follicles, and can also promote oocyte maturation and follicle development by secreting steroid hormones such as E2 and progesterone P4. In this study, Cu^2+^ and TTM were used to treat primary BGCs, and then the Cu^2+^ status in BGCs was determined by detecting intracellular copper concentration and SOD1 activity. The rise and fall in SOD1 and intracellular copper concentrations indicate that BGCs are in a state of high or low copper utilization. The results showed that there was a positive correlation between Cu^2+^ content and hormone secretion of BGCs. Alexander V Sirotkin et al. showed that Cu nanoparticles could promote the release of E2 and P4 from ovarian granulosa cells [24]. S Roychoudhury et al. found that the addition of copper during the in vitro culture of porcine follicle granulosa cells could promote the secretion of steroid hormones [25]. This study also showed that Cu^2+^ promotes the gene and protein expression levels of steroid synthetase in BGCs (Figure 2). This is similar to the results of J C Veltman, where transient treatment of Cu^2+^ in mitochondria promotes a significant increase in cytochrome P450-dependent steroid 11β-hydroxylase activity [26]. In conclusion, the results of the present study indicate that Cu^2+^ promotes the secretion of E2 and P4 from BGCs. StAR is a transporter located in the mitochondrial membrane that facilitates the transfer of cholesterol from the outer mitochondrial membrane to the inner mitochondrial membrane. This process is crucial, as it serves as a key rate-limiting step in the biosynthesis of steroid hormones. CYP11A1 initiates the synthesis of steroid hormones, while 3β-HSD and 17β-HSD function as dehydrogenases.

Transcriptome sequencing results showed that copper level significantly affected the gene expression profile of BGCs. Compared with the control group, the expression of 2773 genes was up-regulated and 1973 genes were down-regulated in the Cu group. In contrast, 213 genes were up-regulated and 94 genes were down-regulated in the TTM group. Further analysis showed that the expression of steroid synthetase genes (such as StAR, CYP19A1, CYP11A1, 3β-HSD, 17β-HSD) was significantly up-regulated in the Cu group, which was consistent with the results of hormone detection, indicating that Cu^2+^ enhanced the hormone secretion ability of BGCs by promoting the expression of steroid synthetase. Functional enrichment analysis of these DEGs showed that the Cu group was significantly enriched in MAPK signaling pathway, TNF signaling pathway and other pathways related to cell proliferation and hormone synthesis, while the TTM group was enriched in herpes simplex virus type 1 infection, the IL-17 signaling pathway and the steroid biosynthesis pathway. Notably, DEGs in the TTM group were significantly enriched in steroid biosynthesis pathways, but their expression levels were generally down-regulated, suggesting that copper deficiency may lead to reduced hormone secretion by inhibiting the expression of genes related to steroid synthesis. This finding is consistent with the results of hormone assays in the TTM group and further validates the critical role of Cu^2+^ in the hormone synthesis of BGCs.

These results suggest that copper may affect the hormone secretion function of BGCs by regulating the MAPK signaling pathway and the expression of related genes. In addition, protein interaction network analysis showed significant interactions between MAPK pathway related genes (such as MAPK3K1, MAPK3K8, MAPK3, etc.) and steroid synthetase genes, further supporting the hypothesis that Cu^2+^ regulates hormone synthesis through the MAPK signaling pathway. Studies have shown that copper ions are transported by copper transporter 1 (Ctr1) to the insides of cells, and enter the mitochondria through specific chaperone proteins (such as COX17) and transporters (such as SCO1/2) to participate in the assembly of respiratory chain enzymes. When copper is out of equilibrium, excess copper significantly increases reactive oxygen species (ROS) production by interfering with the electron transport chain and catalyzing the Fenton reaction. ROS acts as a second messenger to activate downstream ERK1/2, which can phosphorylate transcription factors such as CREB (cAMP Response Element-Binding Protein) and SF-1 (Steroidogenic Factor 1), thereby promoting the transcription of genes such as StAR and CYP19A1 [25,26,27]. In addition, when copper ions are introduced into cells by Ctr1, they are reduced to Cu⁺ by intracellular reductases, and copper chaperones such as CCS, COX17, and ATOX1 transport Cu⁺ to specific target proteins and activate, such as superoxide dismutase 1 (SOD1), cytochrome C oxidase (CCO), and other enzymes in the Golgi apparatus. Copper has also been shown to regulate the phosphorylation activity of metallochaperones such as CCS by interacting with the metal-binding domains of MEK1/2 or ERK1/2 [28]. In conclusion, copper ions can activate MAPK signaling pathways through multiple pathways, but the mechanism of their influence in BGCs hormone secretion remains unclear. The transcriptomic results of this paper suggest that the MAPK signaling pathway is involved in the production of steroid hormones in BGC by copper, so how copper affects the hormone secretion of BGCs through the MAPK signaling pathway will be the focus of our future research.

Taken together, the transcriptome data reveal the molecular mechanism by which Cu^2+^ affects the hormone secretion of BGCs by regulating the expression of genes involved in MAPK signaling pathway and steroid synthesis. These results provide an important molecular basis for a better understanding of the role of Cu in yak reproduction and provide potential targets for the treatment of reproductive disorders associated with Copper deficiency. Although this study revealed the promoting effect of Cu^2+^ on hormone synthesis in BGCs, further in vivo experiments are needed to verify its effectiveness and explore the effects of other potential signaling pathways.

## 5. Conclusions

Cu^2+^ can promote the synthesis of steroid hormones in BGCs by up-regulating the gene and protein expression levels of steroid synthetase, which may be mediated by the MAPK signaling pathway. Although this study has preliminarily explored the law and molecular mechanism of Cu^2+^ regulating BGCs hormone secretion, how Cu^2+^ specifically affects the MAPK pathway to regulate steroid hormone secretion needs to be further explored.

## Figures and Tables

**Table 1:** Primer sequences for qRT-PCR used in the present study.

Gene	Accession No.	Primer Sequences (5′-3′)	Product Size	Tm (°C)
StAR	NM_174189	F: GACACGGTCATCACTCACGA	170 bp	65
		R: ACAAGGTTTCCTGCCACCTC		
CYP11A1	NM_176644	F: TTCAACCTCATCCTGACGCC	149 bp	61
		R: GTGCAAGAGGTGTGGACTGA		
CYP19A1	NM_174305	F: GGTGTCCGAAGTTGTGCCTA	148 bp	65
		R: ACCTGCAGTGGGAAATGAGG		
CYP17A1	NM_174304	F:CCATCAGAGAAGTGCTCCGAAT	80 bp	61
		R: GCCAATGCTGGAGTCAATGA		
3β-HSD	NM_174343	F:AAGACGCAACACCTCAGCAACG	197 bp	60
		R: TGGATCTGCAACACGGGCCAA		
17β-HSD	NM_001102365	F: AAGACGCAACACCTCAGCAACG	100 bp	59
		R: TGGATCTGCAACACGGGCCAA		
β-actin	NM_007393	F: GCTGTGCTATGTTGCTCTAG	117 bp	60
		R: CGCTCGTTGCCAATAGTG		

## Data Availability

The raw RNA-seq reads are available in the NCBI SRA (accession number: PRJNA1250996; https://www.ncbi.nlm.nih.gov/sra/PRJNA1250996) (accessed on 16 April 2025).
